# Longer time to extubation after general anesthesia with desflurane in patients with obstructive respiratory dysfunction: a retrospective study

**DOI:** 10.1186/s40981-021-00443-x

**Published:** 2021-04-30

**Authors:** Eriko Takeyama, Mariko Nakajima, Yukiko Nakanishi, Eizo Amano, Hiromi Shibuya

**Affiliations:** Department of Anesthesiology, National Hospital Organization Osaka National Hospital, 2-1-14, Hoenzaka, Chuo-Ku, Osaka City, Osaka 540-0006 Japan

**Keywords:** Desflurane, Obstructive respiratory dysfunction, Extubation time, Recovery from anesthesia

## Abstract

**Background:**

The prospect of patients with obstructive respiratory dysfunction undergoing surgery has increased with the growth in the elderly population; however, there have been few investigations about the recovery profile from volatile anesthesia. This study aimed to investigate the impact of obstructive respiratory dysfunction on recovery from desflurane anesthesia.

**Methods:**

A retrospective cohort study included patients who underwent orthopedic lower limb surgery between September 2018 and March 2020. Patients were divided into two groups: those whose preoperative forced expiratory volume in 1 s/forced vital capacity ratio was <70% (obstructive respiratory dysfunction group, *n* = 180) or ≥70% (control group, *n* = 45). Time from discontinuation of desflurane to extubation (extubation time) was compared between the two groups. Univariate and multivariable Cox regression models were used to compare odds ratios for prolonged extubation (≥10 min).

**Results:**

A total of 45 patients with obstructive respiratory dysfunction and 180 control patients were eligible for analysis. Extubation time was significantly longer in patients in the obstructive respiratory dysfunction group than those in the control group. In the multivariable Cox model, male sex (HR = 2.00, 95% CI 1.12–3.57; *P* = 0.020) and obstructive respiratory dysfunction (HR = 2.07, 95% CI 1.05–4.08; *P* = 0.036) were associated with prolonged extubation.

**Conclusions:**

This retrospective study indicated that extubation time was longer in patients with obstructive respiratory function than in patients without obstructive respiratory function. Male sex and obstructive respiratory function were factors that contributed to extubation time.

## Background

Obstructive respiratory dysfunction is airflow limitation due to the narrowing of the airways and is characterized by reduced forced expiratory volume in the first second (FEV_1_) with respect to the forced vital capacity (FVC); obstructive respiratory dysfunction is associated with chronic obstructive pulmonary disease (COPD). Almost all surgery patients in Japan undergo spirometry testing as part of routine preoperative examinations regardless of respiratory dysfunction, and we sometimes encounter patients with obstructive respiratory dysfunction immediately before surgery.

Because patients with COPD are known to be at high risk of developing postoperative pulmonary complications [1], careful perioperative management should be performed for patients with obstructive respiratory dysfunction. It is particularly important to wake the patient from anesthesia promptly after surgery to avoid deterioration of postoperative pulmonary function.

For the management of patients with obstructive respiratory dysfunction under general anesthesia, volatile anesthetics are suggested to be useful because of their bronchodilating properties. Among the volatile anesthetics, desflurane is a new fluorinated anesthetic agent with a very low blood-gas partition coefficient, which allows for rapid emergence at the end of surgery [2] and early airway reflex recovery [3]. In addition, desflurane provides faster emergence even in elderly patients, compared with sevoflurane [4], which should indicate its use in this population. Because the prevalence of obstructive respiratory dysfunction is high in the elderly population, desflurane is also efficacious for patients with obstructive respiratory dysfunction.

Conversely, a previous study in a porcine obstructive lung model demonstrated that both uptake and elimination of desflurane were delayed by bronchoconstriction [5]. Moreover, the uptake and elimination of isoflurane, which has a higher blood solubility than desflurane, were less affected by bronchoconstriction, suggesting that the pharmacokinetics of desflurane were likely to be affected by bronchoconstriction due to its low solubility. These data suggest the possibility that patients with obstructive respiratory dysfunction experience a delay in desflurane elimination. However, there are no known reports that assessed the recovery profile from desflurane anesthesia in patients with obstructive respiratory function. Therefore, the objective of our study was to compare the emergence from desflurane anesthesia in patients with and without obstructive respiratory dysfunction.

## Methods

### Patients

Consecutive patients who underwent orthopedic lower limb surgery between September 2018 and March 2020 at National Hospital Organization Osaka National Hospital were evaluated retrospectively. This study included patients who underwent general anesthesia with desflurane. Patients were divided into two groups based on a preoperative spirometry examination. Patients with obstructive respiratory dysfunction were assigned to the obstructive respiratory dysfunction group, and patients without obstructive respiratory dysfunction were assigned to the control group. Obstructive respiratory dysfunction is defined as a preoperative forced expiratory volume in 1 s/forced vital capacity (FEV_1_/FVC) <70%. Exclusion criteria were age younger than 20 years, emergency surgery, body mass index (BMI) ≥35 kg/m^2^, patients managed without tracheal intubation, incomplete patient records, and patients who were not extubated in the operating room.

Anesthesia was induced with fentanyl and/or remifentanil, propofol, and rocuronium in all patients. Patients were then intubated and maintained with desflurane and analgesic fentanyl and/or remifentanil. Anesthesia was maintained with 3 to 6% desflurane. Maintenance of concentrations of desflurane was determined by the attending anesthesiologist. End-tidal CO_2_ pressure was maintained at 35 to 45 mmHg by adjusting the ventilation rate and maximum airway pressure. At the end of the surgery, desflurane was discontinued, and neuromuscular function was restored with sugammadex. When patients regained consciousness by responding to name with spontaneous and smooth respiration, the endotracheal tube was removed. Extubation time was defined as the time from discontinuation of desflurane to extubation.

The study was approved by the institutional review board of the National Hospital Organization, Osaka National Hospital (approval no. 20-151).

### Variables

The following patient data were obtained from medical records: sex, age at the time of surgery, height, weight, BMI, and American Society of Anesthesiologists physical status score. Intraoperative records were reviewed for information regarding operation time, amount of fentanyl administered, maintenance concentration of desflurane, age-adjusted minimal alveolar concentration (MAC) fraction of desflurane, and extubation time (from the desflurane discontinuation to extubation). The age-adjusted MAC fraction was calculated for maintenance concentration of desflurane according to a previously reported equation [6]. To investigate factors related to the delay of extubation after general anesthesia with desflurane, we defined extubation time as ≥10 min as prolonged extubation in this study.

### Statistical methods

Patient baseline clinical characteristics and operative variables are summarized using medians and interquartile ranges for continuous variables and number of patients or percentages for categorical variables. To compare each characteristic between the two groups, the Mann–Whitney *U* test and the chi-square test were used for continuous and categorical variables, respectively.

Logistic regression analysis was used to assess the association between variables attributed to prolonged extubation. Only the meaningful variables (*p* < 0.10) from the univariate analysis were included in the multivariable analysis.

All statistical analyses were performed using JMP Pro software, version 14 (SAS Inc., Cary, NC). All tests were two-tailed, and a *P* value <0.05 was considered statistically significant.

## Results

**Fig. 1:**
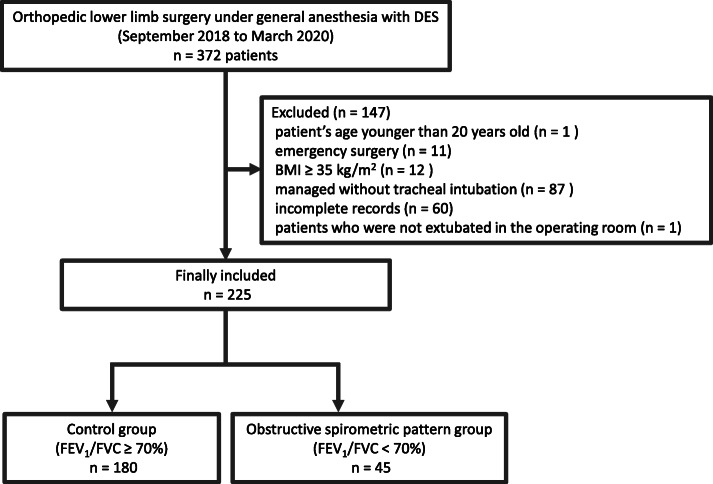
Flow chart for patient’s selection. Flow diagram detailing the selection of patients included in the retrospective analysis. Ninety-one patients were excluded due to non-curative surgery, multiple procedures for the cancer during the study period, incomplete records, and receiving both forms of anesthesia during surgery

A total of 372 patients underwent orthopedic lower limb surgery under general anesthesia with desflurane during the study period. Of these, 147 patients were excluded due to patient age younger than 20 years, emergency surgery, BMI ≥35 kg/m^2^, managed without tracheal intubation, and incomplete patient records (Fig. 1). Patients who were not extubated in the operating room were also excluded. The data from the remaining 225 patients were assessed for the end points, and 45 patients showed obstructive respiratory dysfunction.

Preoperative respiratory function was assessed by spirometry (Table 1). Patients with obstructive respiratory dysfunction showed significantly lower FEV_1_, and FEV_1_/FVC, although there were no differences in FVC and %FVC. Patient characteristics in each group are shown in Table 2. Patients with obstructive respiratory dysfunction were significantly older than patients in the control group (*P* <0.001). There were no other significant differences in patient characteristics, including smoking history.

**Table 1:** Preoperative respiratory function

	Control	Obstructive respiratory dysfunction	
	(*n* = 180)	(*n* = 45)	*P* value
FVC, ml	2765 [2263–3665]	2740 [2195–3350]	0.429
FVC, % predicted	110 [95–124]	104 [95–117]	0.198
FEV_1_, ml	2295 [1740–2845]	1760 [1410–2200]	<0.001*
FEV_1_/FVC, %	78.3 [74.4–82.8]	66.2 [63.1–68.6]	<0.001*

*FVC* forced vital capacity, *FEV*_*1*_ forced expiratory volume in 1 s

*P < 0.05

**Table 2:** Patients’ characteristics

	Control	Obstructive respiratory dysfunction	
	(*n* = 180)	(*n* = 45)	*P* value
Age, yr	73 [60–80]	79 [75–84]	<0.001*
Sex, male/female	77/103	22/23	0.460
Height, cm	156 [148–166]	156 [147–163]	0.598
Weight, kg	60.6 [51.7–69.5]	56.5 [49.7–69.0]	0.329
BMI, kg/m^2^	24.4 [21.9–27.7]	23.1 [21.7–25.3]	0.116
ASA physical status, I/II/III	23/130/27	1/35/9	0.107
Nonsmoker/ex-smoker/current smoker	19/54/106	5/15/24	0.692

*BMI* body mass index, *ASA* American Society of Anesthesiologists

*P < 0.05

Table 3 shows intraoperative demographics of patients in the obstructive respiratory dysfunction group and the control group. Maintenance concentration of desflurane was lower in the obstructive respiratory dysfunction group than in the control group (4.0% vs 5.0%; *P* = 0.002), whereas the age-adjusted MAC fraction was similar between the two groups (*P* = 0.715). No significant difference was observed between the groups in operation time, anesthesia time, and amount of fentanyl used during surgery. The extubation time was significantly longer in the obstructive respiratory dysfunction group than in the control group (9.0 min vs 7.0 min; *P* = 0.014).

**Table 3:** Intraoperative demographics

	Control	Obstructive respiratory dysfunction	
	(*n* = 180)	(*n* = 45)	*P* value
Operation time, min	100 [86–132]	102 [86–117]	0.992
Anesthesia time, min	163 [138–204]	168 [142–188]	0.787
Fentanyl, μg	200 [200–300]	200 [200–300]	0.914
Maintenance concentration of DES, %	5 [4–5]	4 [4–5]	0.002*
Age-adjusted MAC fraction	0.90 [0.84–0.99]	0.88 [0.85–0.99]	0.715
Extubation time, min	7 [5.3–10]	9 [7–12.5]	0.014*
Prolonged extubation (≥ 10min)	47 (26.1)	19 (42.2)	0.034*

*DES* desflurane, *MAC* minimum alveolar concentration

*P < 0.05

The univariate and multivariate logistic regression comparing prolonged extubation time between variants in all patients is shown in Table 4. Male patients and obstructive respiratory dysfunction showed an association with prolonged extubation and were included in multivariable analyses. Both male patients and obstructive respiratory dysfunction were independent risk factors for prolonged extubation time after multivariable analysis (OR 1.96; 95% CI 1.09–3.53; *P* = 0.024 and OR 2.02; 95% CI 1.02–4.02; *P* = 0.045, respectively).

**Table 4:** Univariate and multivariate Cox regression models predicting prolonged extubation time

	Univariate		Multivariate	
	OR (95% CI)	*P* value	OR (95% CI)	*P* value
Sex, male vs female	2.00 (1.12–3.57)	0.020*	1.96 (1.09–3.53)	0.024*
Age ≥ 75 yr	1.28 (0.72–2.27)	0.404		
BMI ≥ 25 kg/m^2^	0.87 (0.48–1.56)	0.641		
ASA, III vs I, II	1.68 (0.80–3.52)	0.172		
Current smoker vs ex-smoker, nonsmoker	1.00 (0.39–2.54)	0.998		
Operation time ≥ 100 min	0.97 (0.55–1.73)	0.925		
Anesthesia time ≥ 160 min	1.52 (0.84–2.75)	0.162		
Fentanyl ≥ 250 μg	1.03 (0.58–1.83)	0.925		
Maintenance concentration of DES ≥ 4.5 %	1.16 (0.65–2.10)	0.614		
Age-adjusted MAC fraction ≥ 1.0	1.04 (0.52–2.07)	0.907		
Obstructive respiratory impairment, yes vs no	2.07 (1.05–4.08)	0.036*	2.02 (1.02–4.02)	0.045*

*OR* odds ratio, *CI* confidence interval, *BMI* body mass index, *ASA* American Society of Anesthesiologists, *DES*, desflurane, *MAC* minimum alveolar concentration

*P < 0.05

## Discussion

We compared the extubation time in patients with and without obstructive respiratory dysfunction after orthopedic lower limb surgery under general anesthesia with desflurane. Patients with obstructive respiratory dysfunction showed significantly longer extubation time than patients without obstructive respiratory dysfunction. Cox regression analysis showed that male sex and obstructive respiratory dysfunction are independent risk factors for prolonged extubation. This is the first known report to evaluate the early recovery from desflurane in patients with obstructive respiratory dysfunction. In contrast, age, operation time, and BMI were not the risk factors for prolonged extubation.

Desflurane is characterized by its low blood/gas partition coefficients, which promote its rapid elimination from the body. An experimental study in an animal model showed that drug-induced bronchoconstriction delayed desflurane elimination [5]. In this animal model, the investigators elucidated that the elimination of desflurane was affected by ventilation-perfusion (V/Q) scatter caused by the shift of ventilation distribution and perfusion dispersion. Furthermore, in the animal model of bronchoconstriction, the elimination of desflurane was more affected due to its low solubility than isoflurane, which is more soluble [7]. These data are consistent with the principle that uptake and elimination of less soluble agents rely more on gas exchange in lower V/Q lung ratios than more soluble agents. In addition, recent clinical studies reported that V/Q scatter caused by general anesthesia is large especially with desflurane, due to its low blood/gas partition coefficient [8]. Therefore, the delay of emergence could be more prominent with desflurane than with other inhalational anesthetics because of the larger V/Q mismatch. A clinical study in patients with COPD indicated that V/Q mismatch was more prominent than expected, even in patients at stage I before FEV_1_ decline [6]. They also showed that low V/Q areas are more prominent than high V/Q areas at the early stages of the disease. These data suggested the possibility that the elimination of desflurane could be delayed due to V/Q mismatch even in patients with slightly decreased FEV_1_/FVC.

The effect of desflurane on respiratory resistance could also affect its elimination. Volatile anesthetics are known to have bronchodilating properties, although recent literature reports conflicting evidence about the effects of desflurane on respiratory resistance. A recent randomized control trial showed that desflurane did not affect respiratory resistance at 1MAC as much as sevoflurane and isoflurane, although 1.5 MAC caused significant increases in respiratory resistance [9]. Conversely, experimental studies on human bronchial tissue showed that desflurane exerted similar relaxant effects on proximal airway smooth muscle as halothane, whereas desflurane was significantly less effective on distal bronchi [10]. The primary region affected in patients with COPD is found in the distal bronchi, which causes expiratory flow limitation. Thus, the obstruction of distal airways, which are less susceptible to the bronchodilating effect of desflurane, may delay the elimination of desflurane. Although the effect of desflurane on respiratory resistance in patients with COPD is still unclear, desflurane was reported to increase the respiratory resistance in patients who smoke [11]. On the other hand, sevoflurane was reported to decrease respiratory resistance in patients with COPD as well as with patients without COPD [12]. However, some patients did not respond to sevoflurane inhalation, with the percentage of those being higher in patients with COPD. These data suggest the possibility that volatile anesthetics including desflurane may not exert bronchodilating effects or even may increase the respiratory resistance in patients with COPD or in smokers. Although the proportion of current smokers was similar between the two groups in this study, desflurane may not show bronchodilating effects or increase respiratory resistance in patients with obstructive respiratory dysfunction, leading to delayed emergence.

Several studies reported that age, BMI, and operation time were associated with prolonged extubation after general anesthesia including general anesthesia induced by volatile agents [13, 14]. It has been shown that emergence and/or recovery from anesthesia are faster with desflurane than with sevoflurane, even in obese patients or elderly patients who are at higher risk for prolonged extubation [3, 4, 15]. However, it is unknown whether the emergence from general anesthesia with desflurane was affected by obesity or older age. Our data indicated that age, BMI, and surgery duration were not associated with extubation time after desflurane anesthesia. The prevalence of COPD is higher in the elderly, and the patients with obstructive respiratory function were older than the control patients in our study. Although maintenance concentration of desflurane was lower in the obstructive respiratory dysfunction group due to the reduced amount of inhalational anesthetic agents required in older patients, age-adjusted MAC was similar between the two groups. Additionally, logistic regression analysis showed that age was not associated with prolonged extubation, indicating that older age of patients in the obstructive respiratory dysfunction group was not the cause of the delay in extubation time.

We also found that male sex was an independent risk factor for prolonged extubation. Previous reports showed that emergence was significantly faster in women after general anesthesia with propofol and volatile anesthetics, including desflurane [16, 17]. An experimental study revealed that females had lower propofol plasma levels and less time to wakening during constant propofol infusion than males [18]. Conversely, it has been reported that there are no pharmacological differences related to sex in the effects of volatile anesthetics on the bispectral index of electroencephalography [19]. Although the underlying mechanisms are still unclear, our data support previous reports showing that the emergence from volatile anesthesia tends to be slower in male patients than in female patients.

Unlike other volatile anesthetics, desflurane has airway-irritating properties at concentrations that exceed 1MAC, which increases the risk of coughing, breath holding, and laryngospasm. Despite these properties of desflurane, studies comparing desflurane with sevoflurane found no differences in the incidence of such respiratory complications [20]. Conversely, these complications were more frequently observed in smokers than in nonsmokers regardless of anesthetic agent. In our study, the proportion of smokers was similar between the two groups, and it is unlikely that the airway-irritating properties of desflurane caused the delay in extubation time.

The clinical relevance of a 2-min slower emergence on outcome in patients with obstructive respiratory dysfunction is debatable. It would only have clinical relevance if it were associated with differences in patient outcomes or resource utilization. Recently, a meta-analysis comparing the early recovery from desflurane and sevoflurane in elderly patients revealed that time to open eyes and extubation was faster in the desflurane group, whereas no significant differences were observed in time to discharge from the recovery room. These data suggest that faster extubation time does not translate to faster recovery. Conversely, patients with obstructive respiratory dysfunction are at risk for airway complications and postoperative pulmonary complications. For such patients, faster emergence and extubation with a secure airway may confer several benefits; thus, it is important to understand the recovery profile from general anesthesia in patients with obstructive respiratory dysfunction. This study suggests the need for additional investigation of recovery profiles after the use of volatile anesthetics in patients with obstructive respiratory dysfunction.

This study has some limitations. First, the study used a retrospective design, and the treatment strategy was not controlled. We did not confirm ventilatory conditions after discontinuation of desflurane, such as the flow rate of fresh gas, respiratory rate, or partial pressure of end-expiratory carbon dioxide, which could affect the elimination of volatile anesthetics. Second, we did not evaluate the actual elimination of desflurane. Thus, longer extubation time in patients with obstructive respiratory dysfunction may not be due to the delay in desflurane elimination. Because the end-tidal volatile agent partial pressure in the presence of V/Q mismatch may not a reliable measure for the arterial blood level, partial pressure in the arterial blood should be measured to assess the elimination of desflurane [8]. Third, it should be considered that in our study, patients with obstructive respiratory dysfunction were not necessarily diagnosed with COPD. All except three patients in the obstructive respiratory dysfunction group were classified as moderate COPD based on the Global Initiative for Chronic Obstructive Lung Disease criteria. Thus, the generalization of our findings may not apply to patients with severe COPD. Fourth, in our study, the patients with obstructive respiratory dysfunction were older than patients in the control group, suggesting they may have comorbidities that would not be expected in the general population. We could not exclude the possibility that some comorbidities could affect the emergence from general anesthesia, although our multivariate analysis showed no association between age and extubation time.

## Conclusions

Obstructive respiratory dysfunction was associated with prolonged extubation time after general anesthesia with desflurane. Our data suggest the possibility that respiratory dysfunction could influence the recovery from volatile anesthetics, including desflurane. Additional prospective studies are needed to understand the early recovery profile from desflurane anesthesia in patients with obstructive respiratory dysfunction.

## Data Availability

The datasets analyzed during the current study are available from the corresponding author on reasonable request.

## Abbreviations

FEV_1_: Forced expiratory volume in the first second
FVC: Forced vital capacity
COPD: Chronic obstructive pulmonary disease
FEV_1_/FVC: Forced expiratory volume in 1 s/forced vital capacity
BMI: Body mass index
MAC: Minimal alveolar concentration
V̇/Q: Ventilation-perfusion
